# Cartilage tympanoplasty for recurrent cholesteatoma using a single sliced cartilage graft: Our experience in 14 ears

**DOI:** 10.1002/ccr3.4799

**Published:** 2021-09-22

**Authors:** Masahiro Komori, Taisuke Kobayashi, Jun Hyodo, Masamitsu Hyodo

**Affiliations:** ^1^ Department of Otolaryngology Kochi Medical School Kochi University Nankoku Japan; ^2^ Department of Otolaryngology Takanoko Hospital Matsuyama Japan

**Keywords:** cartilage tympanoplasty, middle ear aeration, predictor of prognosis, recurrent cholesteatoma, small tympanic cavity

## Abstract

Our procedure may provide a useful alternative in cases where previous surgeries have failed to eradicate the cholesteatoma.

## Graphical abstract

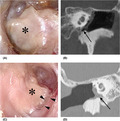

## 1 INTRODUCTION

Few surgical procedures have been proposed for the treatment of recurrent cholesteatoma. Eardrum was totally reconstructed in 14 ears with recurrent cholesteatomas using a single sliced cartilage graft and followed up for more than 5 years. No re‐recurrence of cholesteatoma or complications have been founded.

**FIGURE 1:**
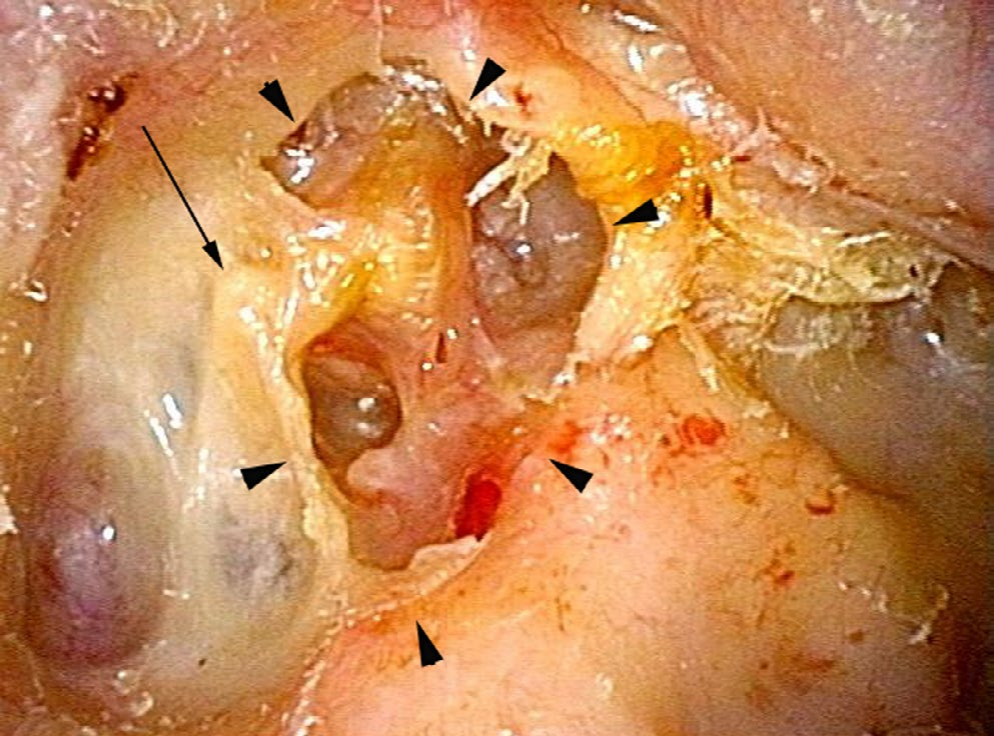
Representative case of recurrent cholesteatoma. The preoperative eardrum finding in case 3 shows a deep retraction pocket (arrowhead), which recurred after canal wall down tympanomastoidectomy. The cholesteatoma debris and matrix have been excised. Arrow indicates the short process of the malleus handle

Postoperative recurrent cholesteatoma due to poor middle ear aeration is a challenging issue in the treatment of cholesteatoma. Canal wall up tympanomastoidectomy (CWUT) obliterates the mastoid bowl, and reconstruction of the scutum of the external auditory canal decreases the risk of recurrent cholesteatoma.^1^, ^2^ Canal wall down tympanomastoidectomy (CWDT) further reduces the incidence of recurrent cholesteatoma or the risk of residual cholesteatoma.^3^, ^4^ Moreover, the posterior wall of the external auditory canal can be reconstructed to preserve the ear anatomy and prevent frequent mastoid bowl debridement. However, in 1.9%–2.6% of ears undergoing CWUT and CWDT, the reconstructed external auditory canal is reabsorbed or deteriorates resulting in retraction.^1^, ^2^, ^3^, ^4^ Moreover, the development of negative pressure in the middle ear may cause the eardrum to retract, leading to a build‐up of the matrix and debris and subsequent recurrent cholesteatoma (Figure 1).

Recurrent cholesteatoma may occur after cartilage tympanoplasty in very poorly aerated ears.^5^, ^6^, ^7^, ^8^ Because cartilage is elastic and has low metabolism, it can withstand negative middle ear pressure and is useful for grafts in middle ears with inadequate blood flow. However, in 2.6%–12% of ears undergoing cartilage tympanoplasty, overlapping portions of cartilage or gaps between pieces of cartilage increase the risk of recurrent cholesteatoma.^7^, ^8^

We performed total reconstruction of the eardrum using a single sliced cartilage graft prepared using a modified microslicing technique^9^ to reduce curling and prevent overlapping or gaps between pieces of cartilage. Few surgical procedures have been described for the treatment of recurrent cholesteatoma, and no studies have investigated procedures in the reoperation context^3^, ^7^, ^8^; that said, Vercruysse et al.^4^ reported one case in which the reconstructed canal wall was resorbed, and the authors closed the epitympanum and reconstructed the canal wall using bone chips and bone pâté. Given the lack of treatment options, our surgical procedure provides a means of obtaining a dry cavity in a specific patient population. Herein, we describe our surgical procedure and present 5‐year follow‐up outcomes.

## 2 METHODS

### 2.1 Ethics approval

The study protocol was approved by the ethics committees of both institutions, and written informed consent was obtained from all subjects prior to surgery.

### 2.2 Subjects

The retrospective case study included 14 ears of 12 patients (average age, 57 ± 14 years) who underwent reoperation between 2006 and 2012 for recurrent cholesteatoma caused by the formation of a deep retraction pocket after CWDT (Table 1). The patients were followed up for at least 5 years (average follow‐up duration, 7.2 ± 1.9 years). An additional two ears of two patients who underwent reoperation during the study period were lost to follow‐up at 0.8 and 2.6 years and excluded from the analysis.

**TABLE 1:** Details of the cases

No.	Sex	Age	Side	Period of follow‐up	Middle ear aeration		Post‐op. Eardrum findings	Air‐bone gaps (dB)	
					Pre‐op.	Post‐op.		Pre‐op.	Post‐op.
1	F	54	L	5.5	(+)	(+)	Good	47.5	30
2	M	67	L	5.6	(+)	(+)	Good	—	—
3	M	66	L	5.7	(+)	(+)	Good	37.5	35
4	F	68	R	5.8	(+)	—	Good	—	—
5	F	42	L	5.8	(+)	(+)	Good	51.25	31.25
6	M	36	R	7.0	(+)	(+)	Good	43.75	22.5
7	M	38	R	11.8	(+)	(+)	Good	—	—
8	M	37	L	5.6	(−)	(−)	Slight ret.	37.5	42.5
9	M	71	R	6.6	(−)	—	Good	scale out	
10	M	64	R	7.0	(−)	(−)	Good	47.5	35
11	M	69	R	8.1	(−)	(+)	Good	—	—
12	M	72	L	8.1	(−)	(+)	TM tube	55	57.5
13	F	46	R	8.6	(−)	(−)	Slight ret.	—	47.5
14	M	69	L	9.7	(−)	(−)	Good	56.25	—

Note

(+) indicates aeration of mesotympanum; (−): no aeration.

Abbreviations: op., operative; ret., retraction; TM, tympanic membrane.

### 2.3 Surgical procedure

After a retroauricular incision was made, the cholesteatoma was removed completely, and the associated inflammatory granulation tissue and malleus and incus, if intact, were removed. Bone was removed to smooth the cavity, and the facial ridge was carefully lowered as much as possible without damaging the facial nerve.

Next, a curved piece of conchal cartilage with bilateral perichondrium large enough to reconstruct the eardrum was harvested with consideration given to the degree of reverse curling that could occur, as reported previously.^9^ Two cartilage plates (0.4–0.5‐mm‐thick), one flat and one markedly curved, were obtained.

**FIGURE 2:**
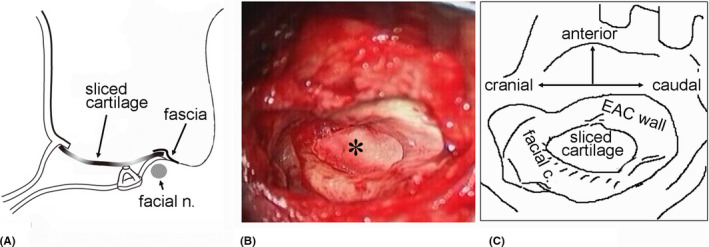
Surgical procedure and representative findings. (A) Schematic of our surgical procedure. (B) Photograph and (C) illustration of a representative surgical finding. A sliced cartilage graft was placed into the meso‐ and infra‐tympanum under the fibrous or bony annulus at the anterior and caudal portions of the graft and onto the horizontal portion of the facial canal (facial c.) and the facial ridge at the posterior and cranial portions of the graft. Facial n., facial nerve; EAC, external ear canal. Asterisks indicate the sliced cartilage

The flat sliced cartilage graft was placed in the mesotympanum and infra‐tympanum with the posterior and cranial portions of the graft placed onto the horizontal segments of the facial canal and facial ridge, respectively (Figure 2). Anterior fibrous annulus present on the eustachian tube was lowered to smooth the transition from the anterior fibrous annulus of the infra‐tympanum to the facial canal. Accordingly, a small tympanic cavity that supports poor middle ear function was reconstructed.

Finally, if necessary, a cartilage columella was placed on the superstructure or footplate of the stapes. A columella was often unnecessary for ears with an intact stapes superstructure because the slight curvature of the sliced cartilage graft meant that it touched the stapes superstructure. Gaps between the graft and the annulus, facial canal, or facial ridge were closed with small pieces of sliced cartilage and then covered with fascia.

### 2.4 Follow‐up study

Follow‐up included microscopic otoscopy, endoscopic otoscopy, and audiometry performed annually. Multi‐slice computed tomography (CT) performed every 1–2 years after surgery was used to assess middle ear aeration and detect recurrent or residual cholesteatoma. The air‐bone gap was assessed using the 4‐tone average (*a* + *b* + *c* + *d*)/4 where *a*, *b*, *c*, and *d* are air‐bone gaps at 0.5, 1, 2, and 4 kHz.

### 2.5 Statistical analysis

Statistical significance was set at *p* < 0.05 and assessed using Fisher's exact test using JMP statistical software (version 11.2.0; SAS Institute).

## 3 RESULTS

**FIGURE 3:**
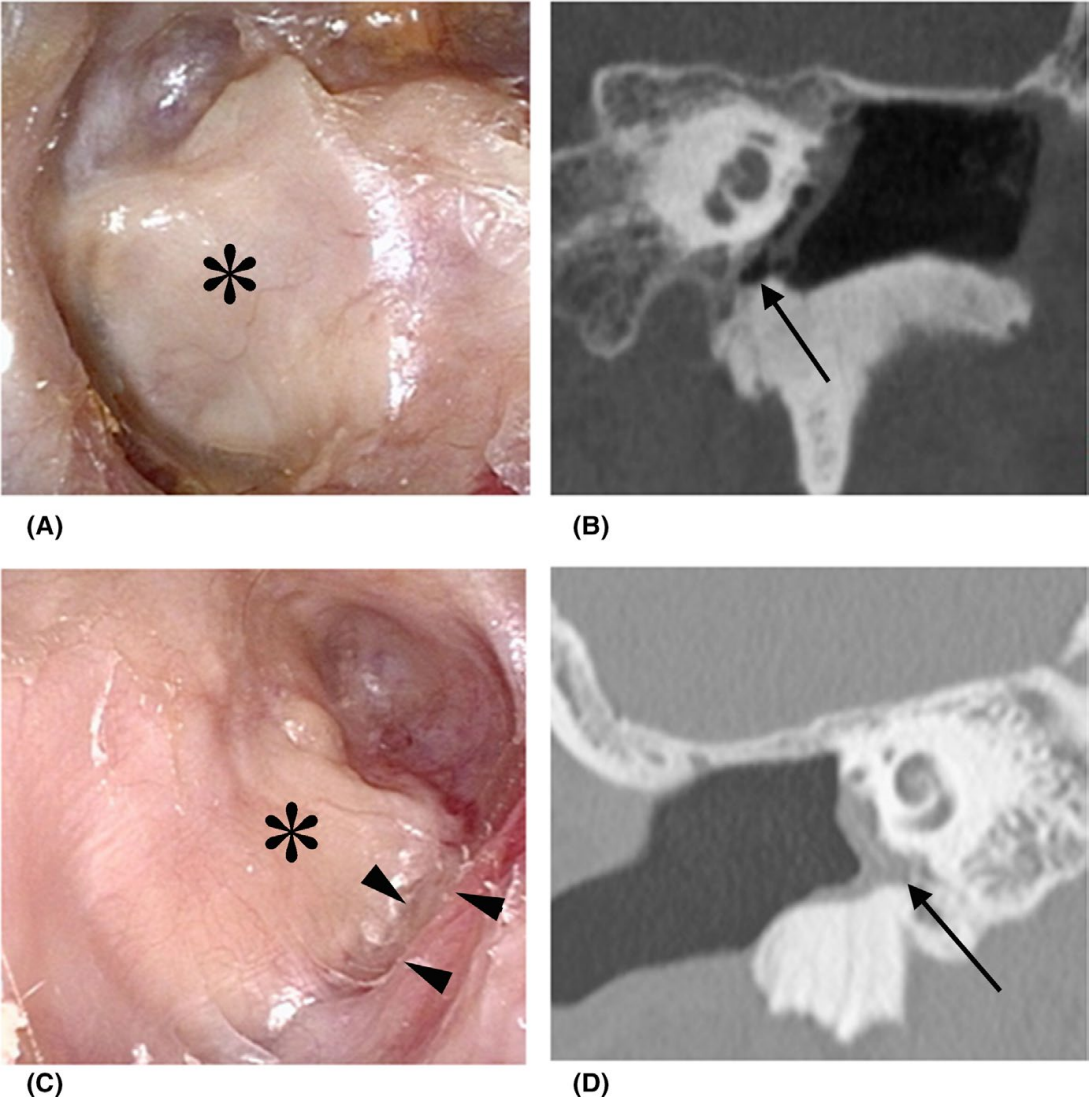
Postoperative findings in two representative eardrums at long‐term follow‐up. (A, B) Photographs of a left ear with an aerated mesotympanum (arrow) show a smooth reconstructed eardrum (asterisk) and tiny bumps between the eardrum and facial ridge at 9 years after surgery. (C, D) Computed tomography findings of a right ear without aeration (arrow) at 8.6 years after surgery show that the eardrum is slightly retracted (asterisk) and the sliced cartilage has become detached from the bony annulus (arrowhead)

Figure 3 shows the postoperative eardrum and CT findings of two representative ears 9 years after surgery. A smooth reconstructed eardrum with tiny bumps between the eardrum and facial ridge can be seen in the ear with an aerated mesotympanum. In contrast, in the ear without aeration, the eardrum was slightly retracted; however, the self‐cleaning function was maintained, and the canal was clear of debris. Additionally, the sliced cartilage had become detached from the bony annulus, possibly due to negative pressure in the middle ear causing the cartilage to retract into the gap that formed.

Preoperatively, seven ears had an aerated mesotympanum and seven ears were without aeration (Table 1). All 14 ears had recurrent cholesteatoma due to deep retraction pockets. Postoperatively, no re‐recurrence was detected in any of the 14 ears after at least 5 years of follow‐up.

Most of the ears (11/14, 79%) had no postoperative complications, such as retraction or perforation. Stratified by preoperative aeration status, all of the ears with an aerated mesotympanum (7/7, 100%) and four ears without aeration (4/7, 57%) were free of complications (*p* = 0.0507, aerated ears vs. ears without aeration). In the remaining three ears without aeration, a tympanic membrane tube was indicated in one ear 3.25 years after surgery, and the eardrums were retracted slightly in two ears; however, they remained clean, without debris. The mean (±SD) pre‐ and postoperative air‐bone gaps were 45.0 ± 5.9 and 29.7 ± 5.3 dB, respectively, in four of seven aerated ears, and 46.7 ± 8.8 and 45 ± 11.5 dB, respectively, in three of seven ears without aeration. The remaining seven ears were excluded from the analysis due to insufficient data. The air‐bone gaps in the aerated ears improved significantly after surgery (*p* = 0.03, preoperative vs. postoperative).

Postoperative changes in aeration were assessed in 12 of 14 ears (two ears were excluded due to missing data) using the most recent CT data. The findings showed that the postoperative levels of aeration were the same as the preoperative levels in all of the aerated ears (6/6, 100%) and in four of six ears without aeration (4/6, 66.6%). In the remaining two ears, including the ear with a tympanic membrane tube, surgery improved the aeration levels to those of the aerated ears.

## 4 DISCUSSION

No re‐recurrence of cholesteatoma was observed in any of the ears in our study at a 5‐year follow‐up. As our study included ears with very poor or no aeration due to recurrent cholesteatoma after CWDT, the findings suggest that our surgical procedure is an effective treatment for recurrent cholesteatoma after previous surgical approaches.

Furthermore, we investigated the usefulness of preoperative middle ear aeration as a predictor of long‐term surgical outcome. No complications, such as retraction or perforation, occurred in the ears with preoperative aeration; moreover, the surgery may have improved hearing levels. In contrast, complications occurred in three ears without preoperative aeration: The eardrum was retracted slightly in two cases, and one case required a tympanic membrane intubation. These findings suggest that preoperative aeration status may provide prognostic information similar to that reported for preoperative aeration status before the second step of staged CWUT.^10^

The indications for our surgical procedure are limited according to recurrent rates of cholesteatoma.^3^, ^4^, ^7^, ^8^ During the study period, we performed 191 tympanoplasties for cholesteatoma at our institutes and, of those, only 14 were performed using the surgical procedure described here (14/191, 7%). Our procedure is a useful second‐line surgical treatment for recurrent cholesteatoma.

### 4.1 Strengths and limitations

The major limitation of our study is the small sample size due to the limited number of suitable cases. Nevertheless, we believe our surgical procedure is useful for the treatment of recurrent cholesteatoma in patients with very poor or no ear aeration.

## 5 CONCLUSIONS

Complete removal of cholesteatoma and morphological preservation of the ear are important goals for the surgical treatment of cholesteatoma. However, in cases where previous surgeries have failed to remove the cholesteatoma, our procedure may provide a useful alternative.

## CONFLICT OF INTEREST

None declared.

## AUTHOR CONTRIBUTION

All authors have been personally and actively involved in substantive work leading to the manuscript, and will hold themselves jointly and individually responsible for its content. MK designed the work and draft the manuscript. MK and JH acquired and analyzed data. TK, JH, and MH revised the manuscript. MK, TK, JH, and MH approved the manuscript and agree to be accountable for all aspect of the work.

## ETHICAL APPROVAL

The study protocol was approved by the ethics committees of both institutions. All procedures performed in studies involving human participants were in accordance with the ethical standards of the institutional and/or national research committee and with the 1964 Helsinki declaration and its later amendments or comparable ethical standards.

## CONSENT

Written informed consent was obtained from all individual participants included in the study.

## Data Availability

The data that support the findings of this study are available from the corresponding author upon reasonable request.
